# Thrombospondin‐2 stimulates MMP‐9 production and promotes osteosarcoma metastasis via the PLC, PKC, c‐Src and NF‐κB activation

**DOI:** 10.1111/jcmm.15874

**Published:** 2020-10-06

**Authors:** Ju‐Fang Liu, Po‐Chun Chen, Tsung‐Ming Chang, Chun‐Han Hou

**Affiliations:** ^1^ School of Oral Hygiene College of Oral Medicine Taipei Medical University Taipei City Taiwan; ^2^ Translational Medicine Center Shin‐Kong Wu Ho‐Su Memorial Hospital Taipei City Taiwan; ^3^ Department of Medical Research China Medical University Hospital China Medical University Taichung Taiwan; ^4^ Department of Biotechnology College of Medical and Health Science Asia University Taichung Taiwan; ^5^ School of Medicine Institute of Physiology National Yang‐Ming University Taipei City Taiwan; ^6^ Department of Orthopedic Surgery National Taiwan University Hospital Taipei City Taiwan

**Keywords:** migration, MMP‐9, osteosarcoma, TSP‐2

## Abstract

Osteosarcoma is an extremely common primary bone malignancy that is highly metastatic, with most deaths resulting from pulmonary metastases. The extracellular matrix protein thrombospondin‐2 (TSP‐2) is key to many biological processes, such as inflammation, wound repair and tissue remodelling. However, it is unclear as to what biological role TSP‐2 plays in human metastatic osteosarcoma. The immunochemistry analysis from osteosarcoma specimens identified marked up‐regulation of TSP‐2 in late‐stage osteosarcoma. Furthermore, we found that TSP‐2 increased the levels of matrix metallopeptidase 9 (MMP‐9) expression and thereby increased the migratory potential of human osteosarcoma cells. Osteosarcoma cells pre‐treated with an MMP‐9 monoclonal antibody (mAb), an MMP‐9 inhibitor, or transfected with MMP‐9 small interfering RNA (siRNA) reduced the capacity of TSP‐2 to potentiate cell migration. TSP‐2 treatment activated the PLCβ, PKCα, c‐Src and nuclear kappa factor B (NF‐κB) signalling pathways, while the specific siRNA, inhibitors and mutants of these cascades reduced TSP‐2‐induced stimulation of migration activity. Knockdown of TSP‐2 expression markedly reduced cell metastasis in cellular and animal experiments. It appears that an interaction between TSP‐2 and integrin αvβ3 activates the PLCβ, PKCα and c‐Src signalling pathways and subsequently activates NF‐κB signalling, increasing MMP‐9 expression and stimulating migratory activity amongst human osteosarcoma cells.

## 1 INTRODUCTION

Osteosarcoma is most commonly diagnosed in the pelvis, or the long bones of the arms and legs.^1^ Current 5‐year survival rates are ~65%, but only 20% in patients with metastatic disease.^2^ The highly invasive nature and metastatic potential of osteosarcoma highlight the importance of understanding how to inhibit metastasis.^3^ Metastatic disease of osteosarcoma frequently manifests as pulmonary metastasis.^4^, ^5^, ^6^ Currently, no therapeutic strategies effectively inhibit the spread of metastasis from the primary osteosarcoma site, particularly to the lungs.

The metastatic cascade represents a series of sequential steps, including invasion, intravasation, migration, attachment, extravasation and growth. It is thought that inhibiting any of these steps should block the entire metastasis process. Matrix metalloproteinases (MMPs) degrade extracellular matrix (ECM) components and are therefore essential for many physiological and pathological processes, including cancer, arthritis and cardiovascular diseases.^7^, ^8^ MMP proteolytic activity affects essential cellular processes including cell proliferation, migration, adhesion, invasion, metastasis and angiogenesis.^9^, ^10^ MMPs at the primary tumour site release soluble factors into the circulation, enabling metastatic growth in distant organs, which allows tumour cells to colonize those sites.^11^ Amongst all MMPs, overexpression of MMP‐9 in particular indicates a higher risk of poor prognosis and poor survival in various cancers.^12^, ^13^ MMP‐9 allows early stage/carcinoma in situ to progress into invasive disease and influences the later development of metastasis. Several cancers express high levels of MMP‐9 expression, such as colon carcinoma,^14^ ovarian carcinoma,^15^ lung carcinoma^16^ and breast carcinoma,^17^ which are associated with metastasis. However, up until now, it has been unclear as to what role of MMP‐9 plays in osteosarcoma progression.

Thrombospondins (TSPs) are large, oligomeric ECM proteins with antiangiogenic functions. These highly conserved, structurally related proteins belong to a glycoprotein family containing five identified members (TSP‐1 to TSP‐5). TSPs are capable of mediating cell‐cell and cell‐matrix interactions, by binding to an array of membrane receptors, other ECM proteins and cytokines. TSPs are capable of modulating cellular development, cell differentiation, cell adhesion and migration, processes that are implicated in the development of atherosclerosis, angiogenesis and oncogenesis. TSP‐2 is found predominantly in chondrogenic and osteogenic processes, in embryonic connective tissue and in adult tissues.^18^ Significant expression of TSP‐2 in developing blood vessels suggests that it is key to the regulation of primary angiogenesis.^19^ TSP‐2 inhibits angiogenesis in endothelial cells and antagonizes vascular endothelial growth factor activity.^20^ Evidence has also revealed the involvement of TSP‐2 in the progression of melanoma, lung and prostate tumour,^21^, ^22^, ^23^ although its role is controversial in tumour progression. While low levels of TSP‐2 expression are associated with poor prognosis in cervical carcinoma^24^ and ovarian carcinoma,^25^ high TSP‐2 expression predicts poor survival for patients with oral cancer^26^ and prostate cancer.^23^ The role of TSP‐2 in the progression of osteosarcoma is largely unknown.

Our findings have identified that overexpression of TSP‐2 predicts a poor prognosis for patients diagnosed with osteosarcoma. Our results also suggest that TSP‐2 potentiates MMP‐9 expression in human osteosarcoma cells and their mobility by regulating the integrin αvβ3/PLC/PKC/c‐Src/NF‐kB signalling transduction pathway.

## 2 MATERIALS AND METHODS

### 2.1 Materials

The secondary antimouse and anti‐rabbit immunoglobulin G (IgG)–conjugated horseradish peroxidases (HRP) and primary antibodies against TSP‐2 (Cat. No. sc‐136238), MMP‐9 (Cat. No. sc‐393859), PLCβ (Cat. No. sc‐136040), PKCα (Cat. No. sc‐8393), c‐Src (Cat. No. sc‐8056), IKKα/β (Cat. No. sc‐7607), IκBα (Cat. No. sc‐1643) and p65 (Cat. No. sc‐8008) were purchased from Santa Cruz Biotechnology. p‐PLCβ^ser537^ (Cat. No. #2481), p‐PKCα/β^Thr638/641^ (Cat. No. #9375), p‐IKKα/β^Ser176/180^ (Cat. No. #2697), p‐IκBα^Ser32/36^ (Cat. No. #9246), p‐c‐Src^ser17^ (Cat. No. #5473) and p‐p65^Ser536^ (Cat. No. #3033) were purchased from Cell Signaling Technology.

Dominant‐negative (DN) mutants of IKKα and IKKβ were kindly provided by Dr H. Nakano (Juntendo University, Tokyo, Japan). The human TSP‐2 recombinant protein was obtained from R&D Systems. The short interfering RNA (shRNA) plasmids were obtained from the National RNAi Core Facility Platform (RNAi Core, Academia Sinica). The inhibitors for PLCβ, PKCα, c‐Src and NF‐κB were obtained from Santa Cruz Biotechnology. All the other chemicals used in molecular biology were obtained from Sigma‐Aldrich.

### 2.2 Cell culture

The human osteosarcoma cell lines (MG63 and HOS), human normal osteoblasts (hFOB) and mouse normal osteoblasts (MC‐3t3‐E1) were bought from the American Type Culture Collection. The osteosarcoma cells were maintained in Eagle's minimum essential medium, hFOB cells were kept in DMEM/F12 medium, and MC‐3t3‐E1 cells were maintained in DMEM, respectively. The 20 mmol/L N‐2‐hydroxyethylpiperazine‐N‐2’‐ethanesulfonic acid, 10% foetal bovine serum (FBS), and 2 mmol/L glutamine, 100 U penicillin/0.1 mg/mL streptomycin (Invitrogen) were added to make complete media. All of the cells were passaged two times a week and were kept at 37°C in a humidified atmosphere containing 5% CO_2_.

### 2.3 Western blot analysis

The cell grown in 6‐well plates and treated with indicated condition described in figure legends section was washed twice with PBS, followed by preparing cell lysates using RIPA lysis buffer. The total proteins were mixed with electrophoresis sample buffer (Cat. No. #161‐0747; Bio‐Rad Laboratories, Inc), boiled and run on SDS‐PAGE, followed by transferred to polyvinylidene fluoride membranes. The blots were then blocked with NAP‐Blocker (G‐Biosciences). The blots were probed with primary antibodies (1:1000) for overnight at 4°C and subsequently incubated with HRP–conjugated secondary antibody (1:2000) against rabbit or mouse IgG for 1 hour at room temperature. The relative information of antibodies used in Western blot was provided in Materials section. The indicated proteins were visualized using ECL^™^ Prime Western Blotting System (Cat. No. RPN2232; GE Healthcare), detected using ImageQuant LAS 4000 bimolecular imager (GE Healthcare Life Sciences). Quantitative analysis of Western blot was conducted using a computing densitometer and ImageQuant (Molecular Dynamics).

### 2.4 Quantitative real‐time polymerase chain reaction

The mRNA expression of indicated genes (Human MMP‐1, ‐2, ‐3, ‐7, ‐9, ‐12 and ‐13, as well as GAPDH) was examined by quantitative real‐time polymerase chain reaction (qPCR) using SYBR Green (KAPA Biosystems) on a StepOnePlus thermocycler (Applied Biosystems) according to the manufacturer's protocol. All of the primers were purchased from Sigma‐Aldrich and used as primers to amplify the target genes. Target gene expression levels were normalized by GAPDH as internal control. The gene expression levels were calculated using the 2^−ΔΔC^ *^t^* method. Each sample was performed with technical triplicate, and the data were conducted from three independent experiments.

### 2.5 Cell migration assay

Transwell chamber migration assay was used to detect cell migratory potential. Briefly, the cells pre‐treated with indicated conditions (as indicated in the figure legends) were seeded to the upper chamber in 200 µL of serum‐free growth medium (10^5^ cells in 24 well chamber with 8.0 µm pore size), followed by provided with 500 µL of serum‐free growth medium to the lower compartment. All of the results were conducted from three independent experiments.

The MG63 (M5) subclone was generated by using Transwell inserts. The MG63 parental cells were placed in upper chamber and performed migration assay in the presence of growth medium contained 10% FBS to the lower compartment. The cell which migrated across membrane of the insert was trypsinized, collected and cultured for 2 days for a second round of selection. After 5 rounds of selection, the subclone was named as MG63 (M5) cells.

### 2.6 Antibody neutralization

The neutralized antibodies against human integrin αvβ3, MMP‐9 or rabbit IgG (Merck KgaA) were used to block biological functions of indicated proteins. After pre‐incubated with neutralized antibodies for 1 hour, the cells were further treated with TSP‐2 for 24 hours. Finally, the cells were collected to perform with Western blot and qPCR analyses.

### 2.7 Immunofluorescence microscopy

The cells were seeded on the 8‐well glass cover slips, followed by treated with indicated conditions which were mentioned in figure legends section. Briefly, cells were washed using PBS, then fixed with 4% paraformaldehyde for 15 minutes, permeabilized with 0.2% Triton X‐100 for 10 minutes and subsequently block with 4% bovine serum albumin for 15 minutes. The cells were incubated with indicated primary antibody (anti‐p65; 1:100) overnight at 4°C, washed with PBS for three times and subsequently incubated with FITC‐conjugated secondary antibody at room temperature for 1 hour. Nuclei were counterstained by DAPI for 5 minutes. Finally, the cells were washed, mounted and monitored by using a Leica TCS SP2 spectral confocal system.

### 2.8 Transfection and reporter gene assay

For luciferase reporter assay, the cells were seeded in 24‐well plates and cotransfected with 1 µg of luciferase plasmid contained conserve NF‐κB binding element, with the negative vector or DN mutants, as indicated in the figure legends. The transfection was performed with Lipofectamine 3000 (Invitrogen). One day post‐transfection, the cells were further incubated with 30 ng/mL TSP‐2 for 24 hours. The cell lysates were collected by lysing cells with reporter lysis buffer (Promega) and collected after centrifugation at 11 000*g* for 2 minutes. A 20 µL portion of the cell lysates was placed into wells of an opaque black 96‐well microplate, and luminescence intensity was measured using the Dual‐Luciferase Assay System (Promega) on a VICTOR^™^ Multilabel Plate Reader (PerkinElmer) according to the manufacturer's protocol. The relative Firefly luciferase activity was measured by normalizing to Renilla luciferase activity.

### 2.9 ChIP assay

Chromatin immunoprecipitation (ChIP) analysis was conducted as previously described.^27^ DNA was immunoprecipitated using anti‐p65 mAb and was further purified. The DNA was extracted after the addition of phenol‐chloroform. The purified DNA pellet was used for PCR analysis. After undergoing PCR, products were resolved using 1.5% agarose gel electrophoresis. UV light was used for visualization. The primers 5′‐CACTTCAAAGTGGTAAGA‐3′ and 5′‐GAAAGTGATGGAAGACTCC‐3′ were used for amplification across the human MMP‐9 promoter region (373 bp including the NF‐κB cluster; GenBank accession no. AF538844).

### 2.10 Establishment of TSP‐2 knockdown stable cell lines

For TSP‐2 knockdown, the shRNA plasmid was purchased from the National RNAi Core Facility Platform (RNAi Core, Academia Sinica). The MG63 cell line was transfected with TSP‐2 shRNA plasmid. An empty vector was used as a negative control. Puromycin (5 μg/mL) was used to screen TSP‐2 shRNA‐expressing cells; surviving cells were used as TSP‐2 knockdown stable cell lines.

### 2.11 In vivo metastasis model

All animal experiments were performed in accordance with a protocols approved by the Institutional Animal Care and Use Committees (with IACUC Approval No: 20160518) of College of Medicine, National Taiwan University (Taipei, Taiwan). Next, 2 × 10^6^ cells were washed and resuspended in 100 μL of PBS and then injected into the lateral tail vein of 5‐week‐old severe combined immunodeficient mice. After 6 weeks, the mice were killed by excess CO_2_. The lungs were photographed, and tumour colonies were manually counted. Subsequently, 10% formalin was used to fix the lungs, which were further embedded in paraffin and processed for haematoxylin and eosin staining.

### 2.12 Immunohistochemistry

Human osteosarcoma tissue microarrays containing 11 cases of normal bone, 9 cases of stage 2 osteosarcoma and 9 cases of stage 3 osteosarcoma were purchased from Biomax. The paraffin‐embedded tissue was sliced, placed on glass slides, rehydrated and incubated in 3% hydrogen peroxide to suppress endogenous peroxidase activity. Next, 3% BSA was prepared and used to block the samples, and subsequently replaced with PBS for incubation. The samples were further incubated at 4°C with a primary mouse polyclonal anti‐human antibody. After overnight incubation, the samples were washed with PBS. After three washes, the samples were incubated with a secondary antibody labelled with biotin. An ABC Kit purchased from Vector Laboratories was used to detect bound antibodies. Next, the samples were stained with chromogen diaminobenzidine. After another wash, the samples were stained with Delafield's haematoxylin. Finally, the samples were dehydrated in alcohols and then mounted in mounting solution.

### 2.13 Statistics

All values are reported as means ± the standard deviation (SD). Significant differences between the experimental groups and controls were assessed using Student's *t* test. The asterisks indicate that the data are significantly different from the controls without TSP‐2 treatment. *Represents *P* < .05 compared with the respective control using one‐way analysis of variance followed by Bonferroni's post hoc test.

## 3 RESULTS

### 3.1 TSP‐2 overexpression supports osteosarcoma cell migration

**FIGURE 1:**
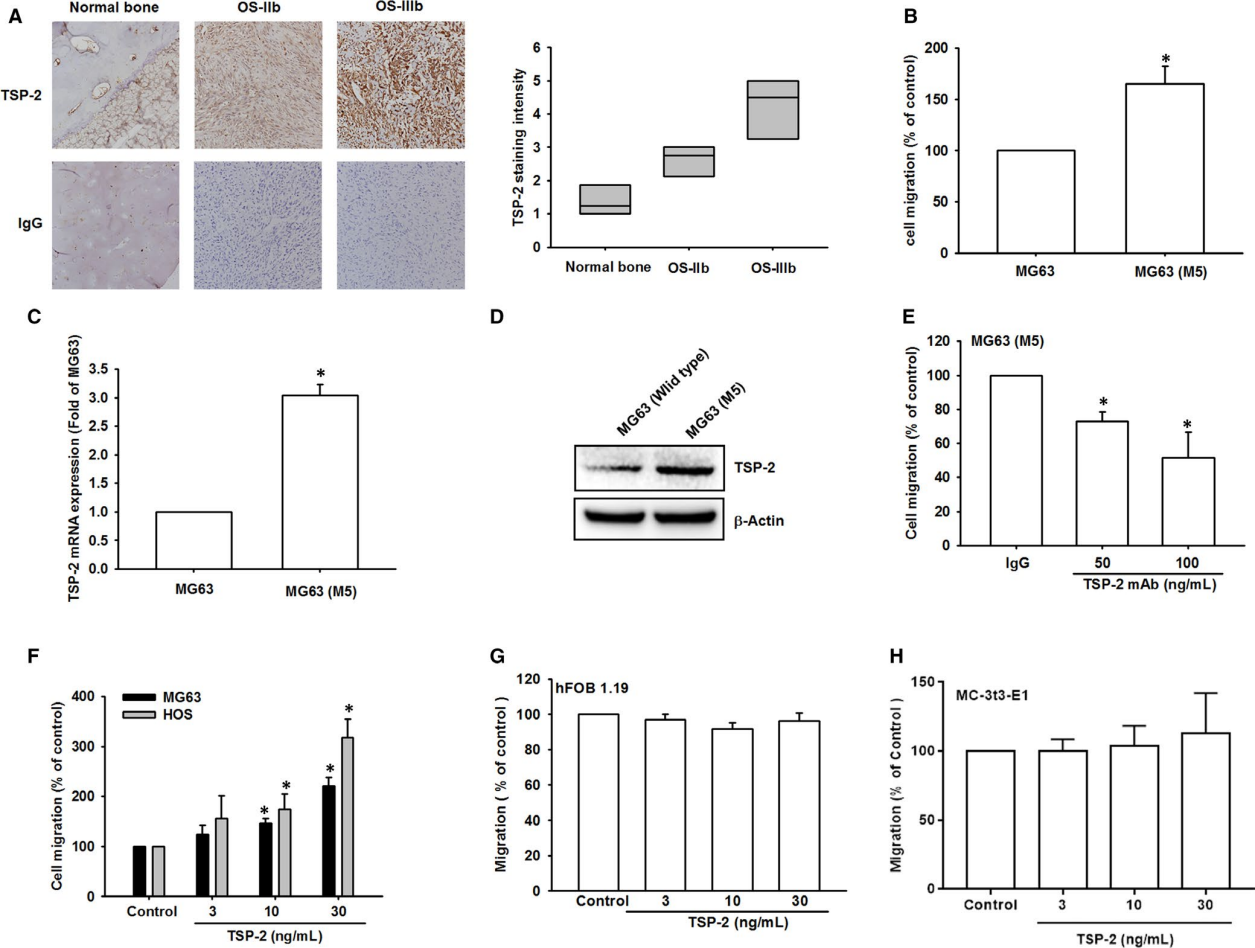
TSP‐2 contributes to the migratory potential of osteosarcoma. A, Osteosarcoma specimens were stained with TSP‐2 antibody. B, The migration abilities of MG63 cells and its migration‐prone subclone (M5) were evaluated by Transwell migration assay. C, D, Total RNA and protein were collected from the MG63 cells and MG63 (M5) cells, and expression level of TSP‐2 was determined using qRT‐PCR and Western blot. E, MG63 (M5) cells were pre‐treated with anti‐TSP‐2 mAb (50‐100 ng/mL) for 24 hours, and then Transwell migration assay was performed to examine cell migration ability after 24 hours. F, G, H, The osteosarcoma cells (MG63 and HOS) and normal osteoblasts (hFOB1.19 and MC‐3t3‐E1) were incubated with TSP‐2 (3‐30 ng/mL) for 24 hours, followed by evaluation of cell migration ability using a Transwell migration assay. The results are expressed as the mean ± SD of triplicate samples. *P < .05 compared with the control group

TSP‐2 overexpression predicts poor survival and metastasis in both urothelial and breast carcinoma.^28^, ^29^ Up until now, no data were available on how TSP‐2 induces metastasis in osteosarcoma. In this study, IHC findings revealed a strong correlation between TSP‐2 concentration and osteosarcoma grades (Figure 1A). Previous report has discussed the migratory potential in various osteosarcoma cell lines.^30^ Therefore, we used HOS cells which showed great migratory ability and MG63 cells which presented lower migratory ability, respectively. Transwell assay data revealed higher levels of TSP‐2 mRNA and protein expression in highly migratory MG63 cell sublines M5 (Figure 1B‐D). In a cell migration assay, M5 cell migration was abolished by pre‐treatment with a TSP‐2‐neutralized antibody abolished (Figure 1E) but dramatically increased by recombinant TSP‐2, as compared with migration of non‐cancerous cells (hFOB 1.19 and MC‐3t3‐E1) (Figure 1F‐H). TSP‐2 appears to influence the migration of osteosarcoma cells.

### 3.2 TSP‐2‐induced stimulation of MMP‐9 expression increases osteosarcoma cell migration

**FIGURE 2:**
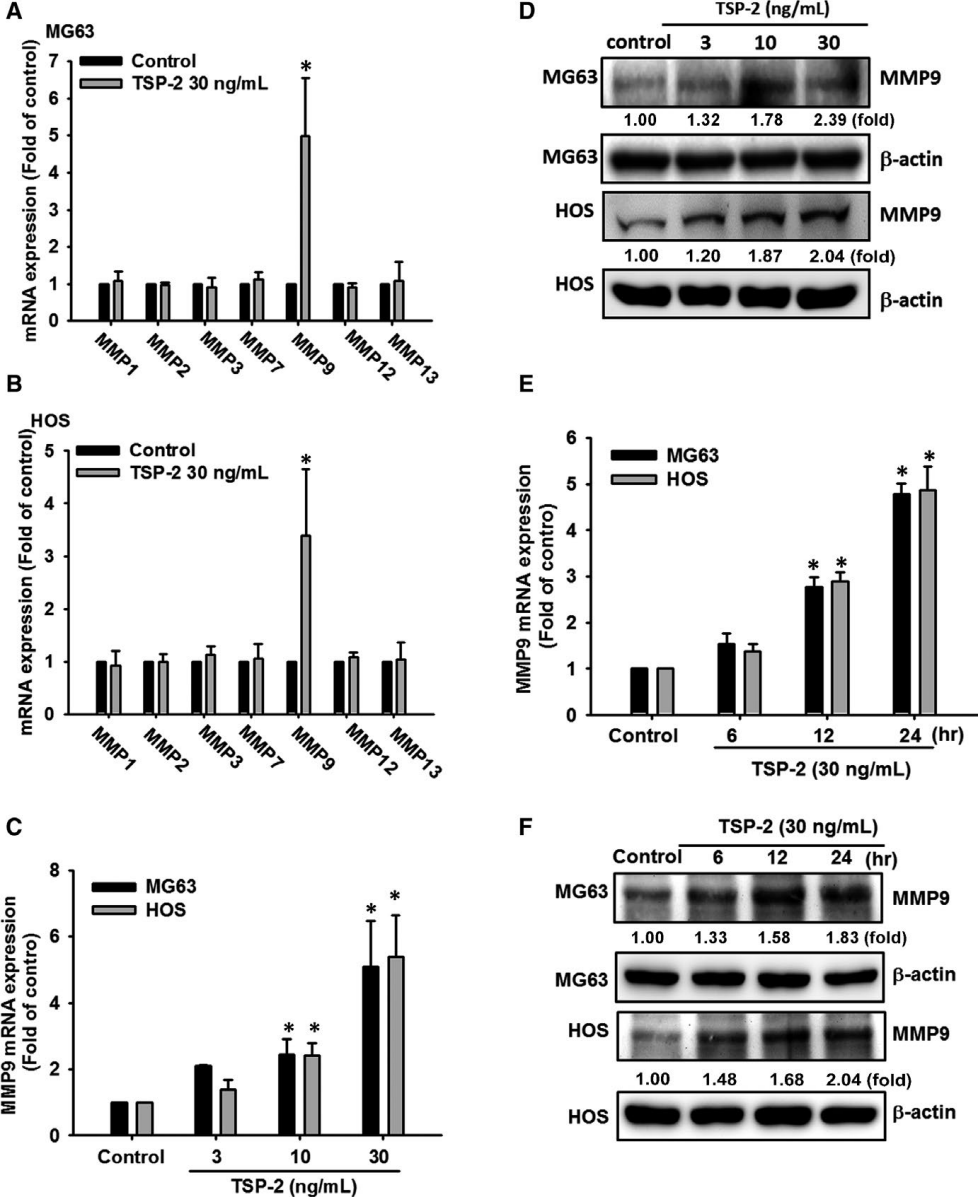
TSP‐2 induces cell migratory potential by MMP9 induction in human osteosarcoma cells. A, B, Osteosarcoma cells (MG63 and HOS) were incubated with TSP‐2 (30 ng/mL) for 24 hours, and the qPCR analysis was conducted to determined levels of MMP‐1, MMP‐2, MMP‐3, MMP‐7, MMP‐9, MMP‐12 and MMP‐13 mRNA expression. C, D, Osteosarcoma cells were incubated with different concentration of TSP‐2 (0‐30 ng/mL) for 24 hours and subsequently examined MMP‐9 expression levels by qPCR and Western blot assay. E, F, Osteosarcoma cells were incubated with TSP‐2 (30 ng/mL) for different time courses (0, 6, 12 and 24 hours), followed by investigation of MMP‐9 expression level through qPCR and Western blot assay. All bars represent the mean ± SD. The asterisks indicate that the data are significantly different from the control without TSP‐2 treatment. *Represents *P* < .05 compared with the respective control using one‐way analysis of variance (ANOVA) followed by Bonferroni's post hoc test

Biological processes that are regulated by TSP‐2 include cellular adhesion, proliferation and angiogenesis, and ECM modelling.^31^, ^32^ Good correlations have been observed between levels TSP‐2 and MMP‐2 expression in prostate cancer,^23^ and TSP‐2‐induced modulation of MMP‐13 expression in lung cancer cells regulates tumour metastasis.^22^ However, it remains uncertain as to the involvement of TSP‐2 in MMP expression in osteosarcoma cells and whether TSP‐2 could help to predict osteosarcoma prognosis. To identify the mediator of TSP‐2‐promoted osteosarcoma migration, we examined levels of MMP‐1, MMP‐2, MMP‐3, MMP‐7, MMP‐9, MMP‐12 and MMP‐13 mRNA expression following TSP‐2 stimulation (Figure 2A,B). We found that only MMP‐9 mRNA was substantially increased. Interestingly, we observed dose‐dependent (Figure 2C,D) and time‐dependent (Figure 2E,F) up‐regulation of MMP‐9 mRNA and protein expression. The expression patterns of TSP‐2 and MMP‐9 were also similar amongst osteosarcoma cell lines and normal osteoblast, with greatest expression in HOS cells (Figure S1). In summary, these data reveal the association of MMP‐9 in TSP‐2‐promoted cell migration.

### 3.3 Integrin ανβ3 contributes to TSP‐2‐induced effects on cell migratory potential and induction of MMP‐9 in osteosarcoma cells

**FIGURE 3:**
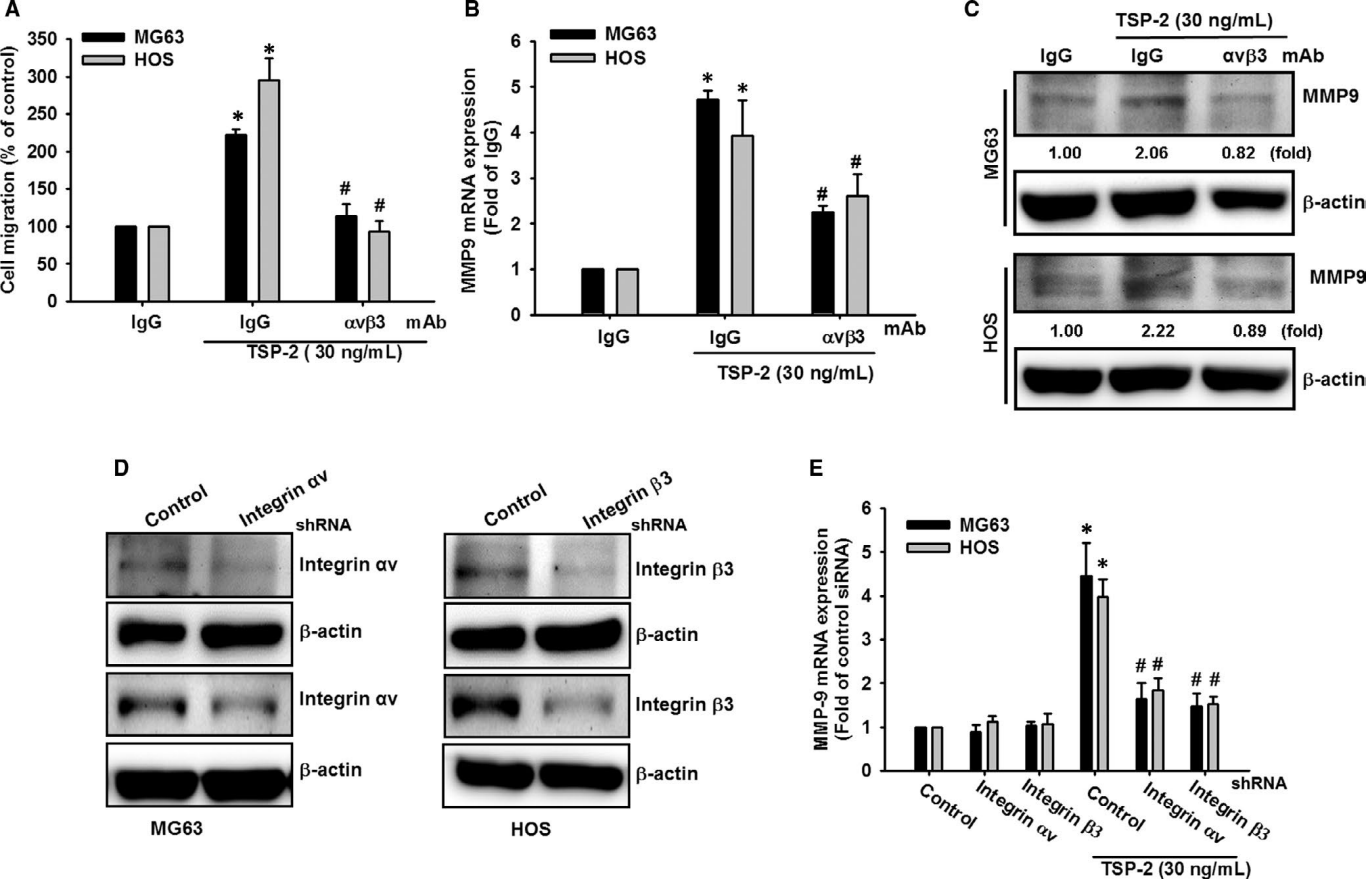
Integrin αvβ3 is responsible for TSP‐2‐promoted MMP‐9 expression and cell migratory potential in osteosarcoma cells. A‐C, Osteosarcoma cells (MG63 and HOS) were pre‐treated with integrin αvβ3 neutralized mAb (3 μg/mL) or IgG isotype control for 30 minutes and then stimulated with TSP‐2 (30 ng/mL) for 24 hours. A, Cell migratory potential was determined by a Transwell migration assay. B, C, The expression levels of MMP‐9 were determined by qPCR and Western blot. D, E, Osteosarcoma introduced with shRNA plasmids which against integrin αv, β3 shRNA or control vector were incubated with TSP‐2 (30 ng/mL) for 24 hours, followed by evaluation of expression levels of MMP‐9 by qPCR. The results are expressed as the mean ± SD of triplicate samples. *P < .05 compared with the control group, and ^#^ *P* < .05 compared with TSP‐2 treatment

The biological functions including adhesion and migration regulated by TSP‐2 have been reported,^33^, ^34^ with interaction between arginine‐glycine‐aspartic acid sequence of TSP‐2 and integrin ανβ3. We therefore examined the effect of integrin ανβ3 neutralizing antibody to investigate the effect of integrin ανβ3 neutralization in TSP‐2‐induced stimulation of osteosarcoma cell migratory potential and MMP‐9 expression. The cells pre‐incubated with integrin ανβ3 antibody substantially abrogated cell migration in response to TSP‐2 treatment (Figure 3A,B) and induction of MMP‐9 (Figure 3C). Moreover, transfection of osteosarcoma cells with integrin αv and integrin β3 shRNA dramatically abolished cell migration in response to TSP‐2 treatment (Figure 3E). In summary, integrin αvβ3 effectively modulates TSP‐2‐induced MMP‐9 expression and migratory potential in osteosarcoma cells.

### 3.4 The PLC, PKC and c‐Src signal pathways contribute to cell mobility and MMP‐9 expression in response to TSP‐2 treatment in osteosarcoma cells

**FIGURE 4:**
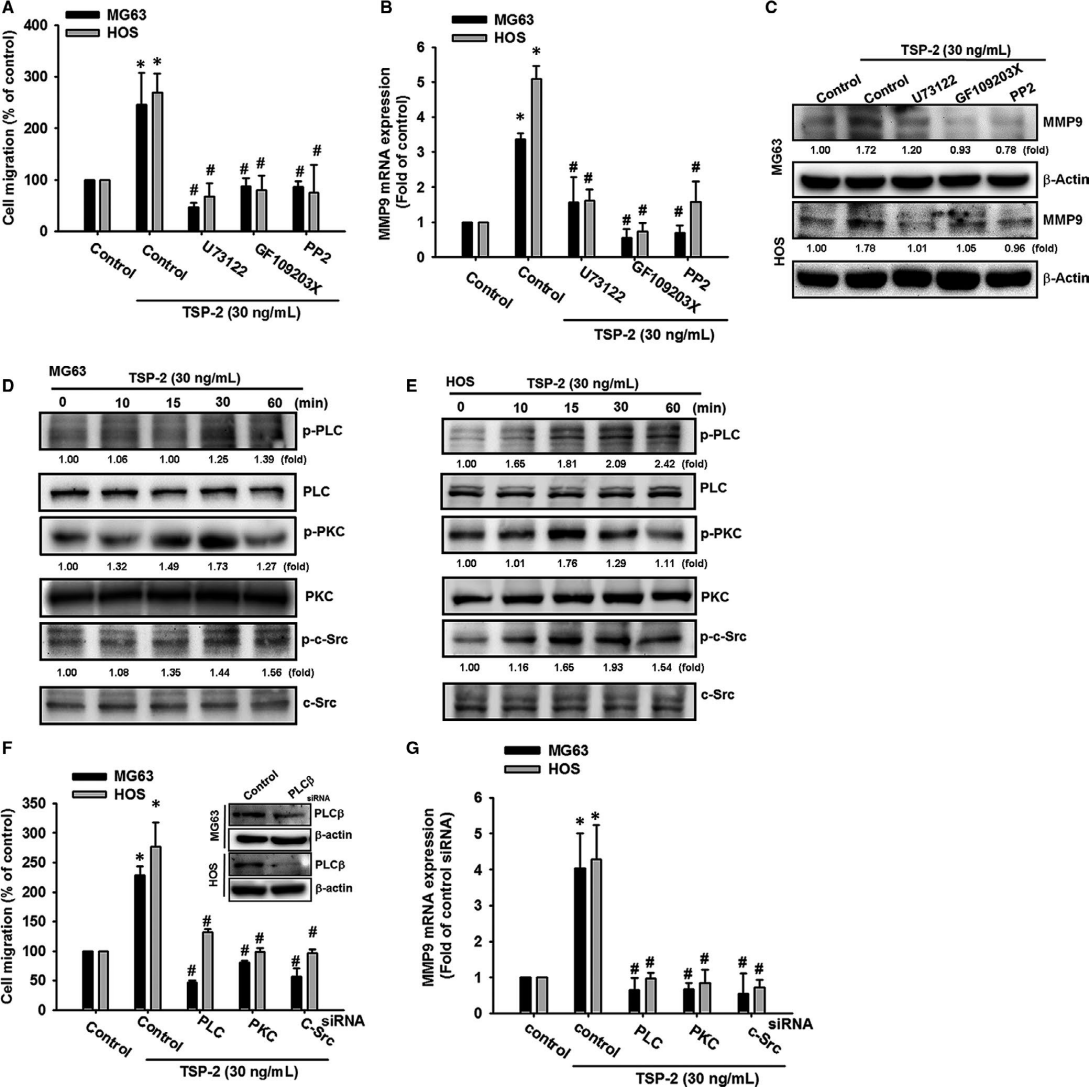
PLC/PKC/c‐Src signalling transduction mediates MMP‐9 expression and cell migratory potential after TSP‐2 stimulation in human osteosarcoma cells. A, Osteosarcoma cells (MG63 and HOS) were pre‐treated with PLC (U73122; 0.5 μmol/L), PKC (GF109203X; 3 μmol/L) and c‐Src (PP2; 5 μmol/L) inhibitors for 30 minutes, followed by TSP‐2 (30 ng/mL) stimulation for 24 hours. Cell migration was measured by Transwell assay. B, C, Cells were treated as described in (A). Total RNA and protein were extracted, and MMP‐9 expression was assessed by qPCR and Western blot. D, E, Cells were incubated with TSP‐2 for 10, 15, 30 and 60 minutes, and PLC, PKC and c‐Src phosphorylation was determined by Western blot. F, Cells were transfected with a control siRNA or PLC siRNA, PKC siRNA or c‐Src siRNA for 24 hours and subsequently incubated with TSP‐2 (30 ng/mL) for 24 hours. Cell migratory potential was measured using Transwell migration assays. G, Cells were treated as described in (D), and MMP‐9 expression was investigated by qPCR. Untreated cells were used as controls (set to 100%); the data are shown as multiples of that. Results are shown as the means ± SD (n ≥ 3; *P < .05 compared with untreated controls; ^#^ *P* < .05 compared with the TSP‐2‐treated group)

It is known that integrin signalling triggers several pathways, including the PLC/PKC/c‐Src pathway,^35^, ^36^, ^37^ and our data indicated that TSP‐2 exhibited its biological activity via integrin αvβ3 receptors. We therefore sought to determine whether the PLC, PKC and c‐Src pathways play a role in TSP‐2‐induced migratory potential in osteosarcoma cells. The cells were administered PLC, PKC and c‐Src chemical inhibitors, prior to TSP‐2 treatment. All three inhibitors significantly blocked migratory potential and induction of MMP‐9 after TSP‐2 incubation (Figure 4A‐C). Meanwhile, TSP‐2 treatment increased expression of phosphorylated forms of PLC, PKC and c‐Src (Figure 4D,E). Furthermore, transfecting cells with PLC siRNA, PKC siRNA and c‐Src siRNA suppressed TSP‐2‐promoted cell migratory potential and MMP‐9 expression (Figure 4F,G). Finally, pre‐incubation with integrin ανβ3 neutralizing antibody abrogated PLC, PKC and c‐Src activation in response to TSP‐2 in osteosarcoma cells (Figure S2). Therefore, this evidence indicates that PLC, PKC and c‐Src signal pathways are responsible for migratory potential after TSP‐2 treatment.

### 3.5 TSP‐2 enhances osteosarcoma cell migratory potential via NF‐κB transcriptional activation

**FIGURE 5:**
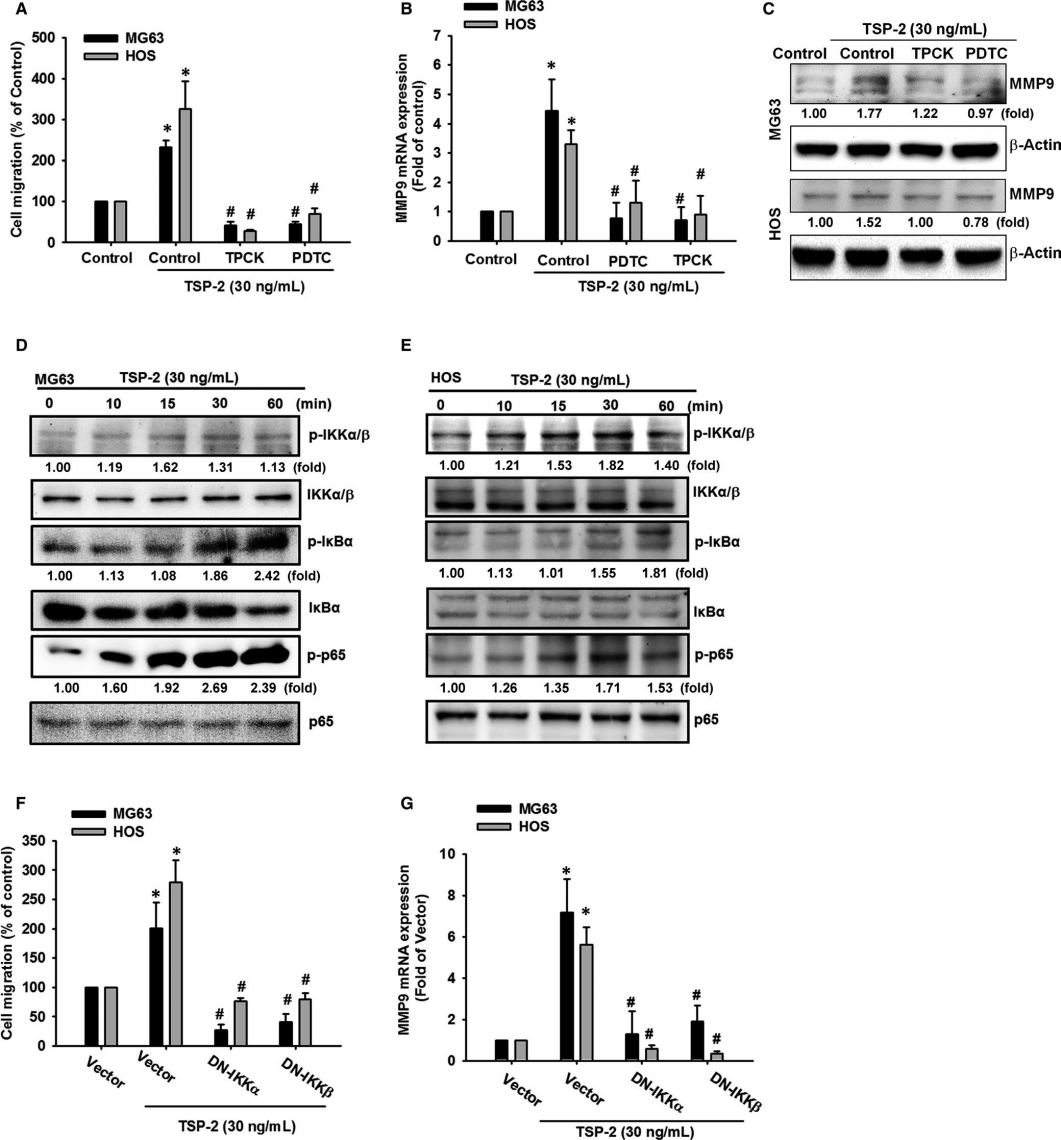
NF‐κB transcription factor participates MMP‐9 expression and cell migratory potential in response to TSP‐2 treatment in human osteosarcoma cells. A‐C, Osteosarcoma cells (MG63 and HOS) were pre‐treated with PDTC (5 μmol/L) or TPCK (5 μmol/L) for 60 minutes and subsequently incubated with TSP‐2 (30 ng/mL) for 24 hours. A, Cell migratory potential was measured by Transwell assays. B, C, MMP‐9 expression levels were evaluated by Western blot and qPCR. D, E, Osteosarcoma cells were incubated with TSP‐2 for different time intervals (10, 15, 30, 60 and 120 minutes), followed by evaluation of p65, IKKα/β and IκBα phosphorylation by Western blot. F and G, The control vector, DN‐IKKα, or DN‐IKKβ plasmids were introduced to osteosarcoma cells for 24 hours, followed by stimulation of TSP‐2 (30 ng/mL) for 24 hours. F, Cell migratory potential was examined by the Transwell migration assay. G, MMP‐9 expression was evaluated by qPCR. Untreated cells were used as controls (set to 100%); the data are shown as multiples of that. Results are shown as the means ± SD (n ≥ 3; *P < .05 compared with untreated controls; ^#^ *P* < .05 compared with the TSP‐2‐treated group)

The commonly observed association between NF‐κB activity, cancer cell migration and invasion^38^, ^39^ led us to investigate the possible involvement of the NF‐κB transcription factor in TSP‐2‐promoted effects in osteosarcoma cells. Pre‐treating the cells with the NF‐κB signal cascade inhibitors (pyrrolidine dithiocarbamate; PDTC and 6‐(1‐tosylamido‐2‐phenyl) ethyl chloromethyl ketone; TPCK) reversed TSP‐2‐induced cell mobility (Figure 5A‐C). The components of NF‐κB signal cascade such as p65, IKKα/β and IκBα were stimulated by TSP‐2 treatment in osteosarcoma cells (Figure 5D,E).

**FIGURE 6:**
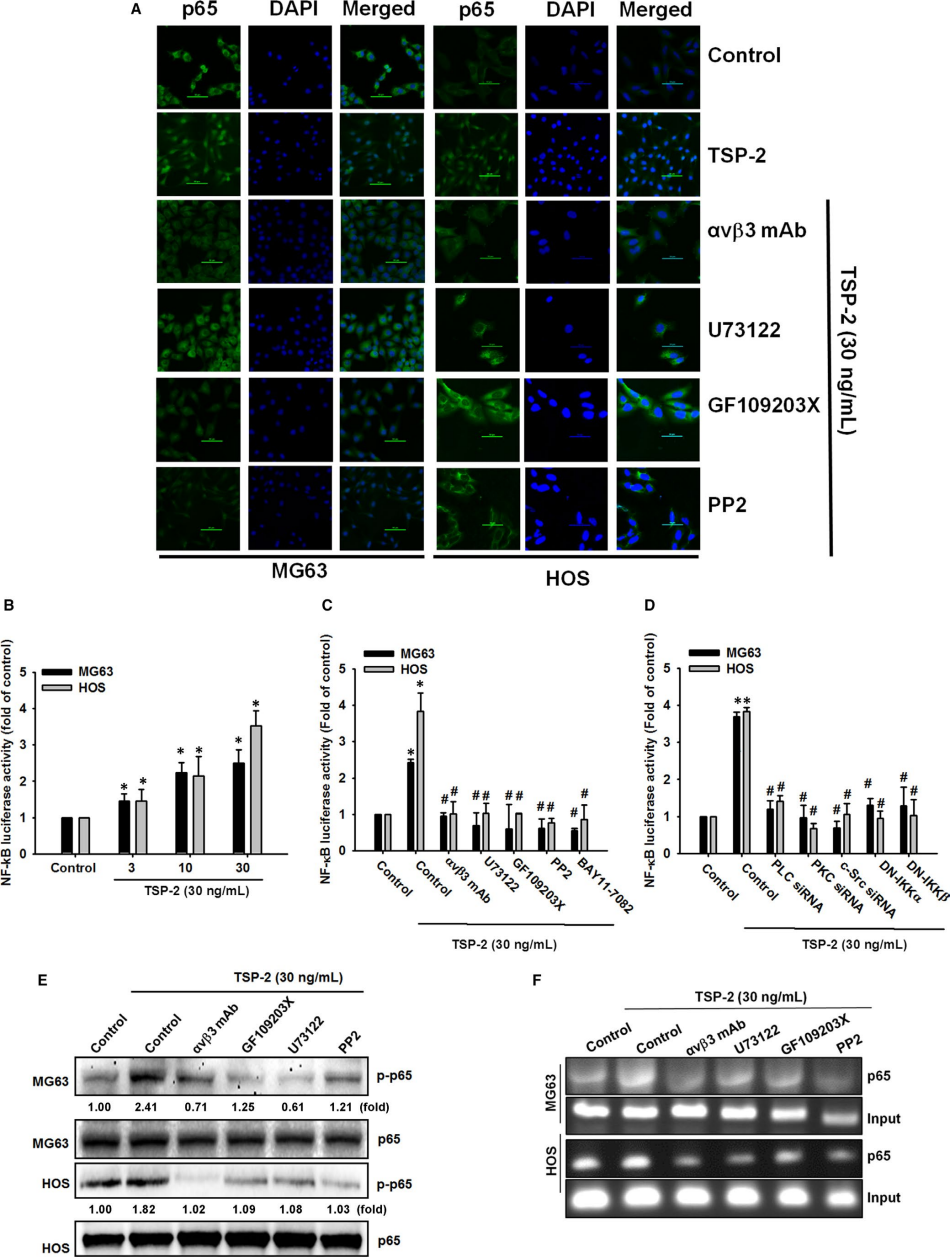
NF‐κB transcriptional activation is required for TSP‐2‐promoted MMP‐9 expression and cell migratory potential in human osteosarcoma cells. A, Osteosarcoma cells were pre‐treated with integrin αvβ3 mAb, U73122 (0.5 μmol/L), GF109203X (3 μmol/L) and PP2 (5 μmol/L) for 30 minutes and subsequently incubated with TSP‐2 (30 ng/mL) for 1 hour. The p65 antibody (green) was used to monitor nuclear translocation of NF‐κB by immunofluorescence stain. DAPI (blue) was used as counterstain, and representative microscopic results were photographed. (Scale bar = 50 μm). B, NF‐κB promoter reporter plasmid was introduced to osteosarcoma cells for 24 hours and then incubated with various concentrations of TSP‐2 (0‐30 ng/mL) for 24 hours, followed by assessment of luciferase activity. C, NF‐κB promoter reporter plasmid was introduced to osteosarcoma cells for 24 hours and then treated with integrin αvβ3 mAb, U73122, GF109203X, PP2 and BAY11‐7082 for 30 minutes. The cells were further treated with TSP‐2 (30 ng/mL) for 24 hours, and luciferase activity was determined. D, Osteosarcoma cells were cotransfected with NF‐κB promoter reporter plasmid and PLC siRNA, PKC siRNA and c‐Src siRNA, as well as DN‐IKKα or DN‐IKKβ plasmids for 24 hours, and subsequently stimulated with TSP‐2 (30 ng/mL) for 24 hours and luciferase activity was determined. E, Osteosarcoma cells were treated as described in Figure 5C, followed by investigation of p65 and IKKα/β phosphorylation by Western blot. F, Cells were pre‐treated with integrin αvβ3 mAb, U73122 (0.5 μmol/L), GF109203X (3 μmol/L) and PP2 (5 μmol/L) for 30 minutes and subsequently stimulated with TSP‐2 (30 ng/mL) for 1 hour. The p65 antibody was used to confirm binding of NF‐κB to DNA element by using ChIP. One per cent of immunoprecipitated chromatin was used as loading control (input). Untreated cells were used as controls (set to 100%); the data are shown as multiples of that. Results are shown as the means ± SD (n ≥  3; *P < .05 compared with untreated controls; ^#^ *P* < .05 compared with the TSP‐2‐treated group)

Treatment with inhibitors and DN mutants which targeting NF‐κB pathway components also inhibited cell migratory potential and MMP‐9 expression in response to TSP‐2 incubation (Figure 5F,G). The p65 nuclear translocation revealed TSP‐2‐induced NF‐κB cellular activity, which was abrogated by pre‐incubation with PLC, PKC and c‐Src inhibitors (Figure 6A). The TSP‐2‐promoted induction of NF‐κB promoter activity was examined by luciferase reporter assay (Figure 6B). Meanwhile, pre‐treatment with inhibitors or pre‐transfected with DN mutants against NF‐κB pathway obviously prevented transcriptional activation of NF‐κB, which was stimulated by TSP‐2 incubation (Figure 6C,D).

When the cells were pre‐incubated with integrin αvβ3 neutralized antibody, PLC, PKC and c‐Src inhibitors, p65 nuclear translocation and phosphorylation was blocked (Figure 6E), while results of a ChIP assay indicated that the pathway inhibitors blocked NF‐κB from binding to its DNA element (Figure 6F). This evidence proposes that NF‐κB transcription factor activation contributes to TSP‐2‐promoted MMP‐9 expression and cell migratory potential, as well as PLC/PKC/c‐Src/NF‐κB signalling.

### 3.6 *TSP‐2 knockdown abolishes lung metastasis* in vivo

**FIGURE 7:**
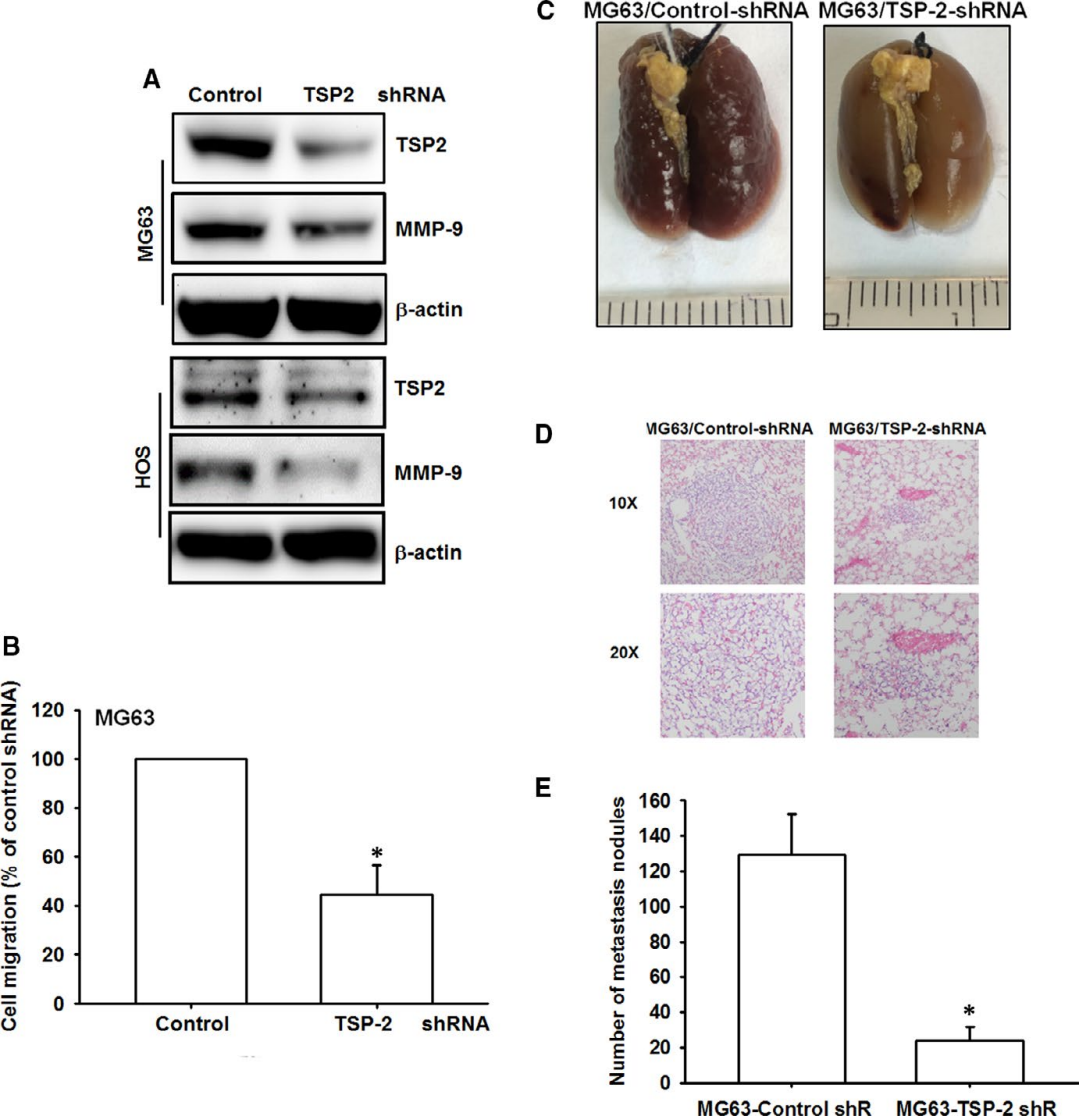
TSP‐2 knockdown suppresses lung metastasis in vivo. (A and B) TSP‐2 shRNA and vector control were introduced to MG63 cells to generate stable clone. A, The expression levels of TSP‐2 and MMP‐9 were assessed by Western blot. B, Migration potential of MG63 cells stable clones was determined using the Transwell migration assay. C, The MG63 stable clones were implanted by tail vein injection to induce pulmonary metastases, and the mice were killed after 28 days for evaluation of lung metastatic nodules. D, Haematoxylin and eosin stain of lung metastatic nodules in mice implanted with MG63 stable clones; representative images were captured at 100× magnification, and the scale bars represent 250 μm. E, The lungs were collected from killed mice were perfused with 10% paraformaldehyde. The number of lung metastatic nodules was monitored under a dissecting microscope. Results are shown as the means ± SD. The results are expressed as the mean ± SD of triplicate samples. *P < .05 compared with the control group

We observed declines in TSP‐2 and MMP‐9 expression in clones stably expressing TSP‐2 shRNA (Figure 7A) and a reduction in cell migration ability in clones stably expressing TSP‐2 shRNA (Figure 7B), compared with vector‐only control clones. MG63 cells stably expressing a shRNA vector or control vector cells were then injected into mouse tail veins; all animals were killed 28 days later. The mean number of lung metastatic nodules in tumour‐bearing mice was significantly reduced by TSP‐2 knockdown (Figure 7C‐E). Our evidence demonstrates conclusively that TSP‐2 knockdown reduces the migratory ability of osteosarcoma cells and lung metastasis in an osteosarcoma animal model.

## 4 DISCUSSION

The multifunctional matricellular glycoprotein, TSP‐2, is implicated in mechanisms relevant to tumorigenesis^40^ and in antiangiogenic activity associated with tumour progression.^41^, ^42^ Cervical cancer tissue, especially with lymphatic metastatic involvement, demonstrates increased levels of miR‐221‐3p and TWIST2 expression and decreased levels of TSP‐2 expression, which implies that TWIST2 stimulates miR‐221‐3p expression and thus abrogates TSP‐2‐promoted metastasis in cervical cancer.^43^ In human malignant melanoma, TSP‐2 suppresses haematogenous metastasis through microenvironment modification, including plasminogen activator inhibitor up‐regulation and antivascularization.^21^ In contrast, TSP‐2 expression is up‐regulated in mRNA, and increased TSP‐2 expression in colorectal cancer is associated with poor overall survival.^44^ High TSP‐2 expression enhances tumour growth and accelerates tumour cell metastasis in colorectal cancer.^45^ In 2017, Chen et al used miR‐376c mimic stimulation to suppress TSP‐2‐induced MMP‐2 expression and cell motility, and demonstrated that administering TSP‐2 knockdown to prostate cancer cells abolished osteolytic metastasis in vivo,^23^ revealing that TSP‐2 has dual functionality in cancer progression. Our data demonstrate higher TSP‐2 expression in osteosarcoma tissue than in normal bone tissue, and also confirm TSP‐2 has capacity to increase MMP‐9 expression, by which to enhance cell migratory potential in osteosarcoma disease.

MMPs, a family of calcium‐ and zinc‐dependent proteinase,^46^ are associated with the survival and expansion of cancer cells, regulating their growth through measures including the release of cell membrane–bound precursors of various growth factors, modulating growth factor bioavailability and indirectly regulating the proliferative signals of integrins. In 2008, Nakamura et al demonstrated that TSP‐2 inhibits cell invasion by down‐regulating MMP‐9 and urokinase‐type plasminogen activator (µPA) activity in pancreatic cancer cell lines.^47^ Other research has indicated lower levels of MMP‐2 and MMP‐9 expression and higher levels of MMP‐12 and MMP‐17 expression in transformants overexpressing the TSP‐2 gene compared with a vector control transformant.^47^, ^48^ It is speculated that the TSP‐2 gene may modulate the progression of cancer by regulating the MMP family and thereby degrading cancer stroma.^49^ Interestingly, an investigation into the role played by MMPs in human osteosarcoma cell lines, xenografts and biopsies found low detectable amounts of MMP‐9 in the U2OS cell line only and MMP‐9 mRNA was below the level of detection in the xenografts, whereas MMP‐9 was not only found in 11/12 biopsies, but was present with very high expression in 5 of those 11 biopsies.^3^ When the researchers examined the invasive properties and gelatinolytic activity of the cell lines, they found that U2OS cells were the most invasive and secreted the highest amounts of MMP‐2 and MMP‐9. Those study researchers suggest that as osteosarcoma cell invasion was significantly reduced by two different MMP inhibitors, MMPs appear to be critically important for the invasiveness of human osteosarcoma cells.^3^ Recently, TSP‐2 has been proved to promote metastasis by induction of MMP‐2 expression.^23^ In line with this finding, our study has revealed high levels of MMP‐9 expression in osteosarcoma bone tissue. We also found positive correlation between TSP‐2 and MMP‐9 in osteosarcoma cells. Furthermore, TSP‐2 appeared to promote MMP‐9 expression by which promotes cell migratory potential in osteosarcoma cells.

Integrins are TSP‐2 receptors that are capable of mediating tumour cell‐ECM adhesion and facilitate the connection between the adhesive substrate and cellular signalling required by cells for proliferation, migration and invasion. It is suggested that integrin α_V_β_3_ regulates biological functions executed by TSP‐2, such as angiogenesis and cell migratory potential.^50^ Integrin α_V_β_3_ has been proposed to deal with tumoural angiogenesis.^51^, ^52^ Notably, elevated expression of integrin αvβ3 is associated with metastasis.^53^ Furthermore, targeting integrin α_V_β_3_ could ameliorate lung metastasis in vivo as previous described,^54^ suggesting the pivotal role of integrin αVβ3 in osteosarcoma metastasis. The evidence in this study suggests that TSP‐2 stimulates cell migratory potential through integrin α_V_β_3_, whether TSP‐2 modulates angiogenetic effects in osteosarcoma should be elucidated in the future.

In conclusion, we determined the following: (a) higher level of TSP‐2 was found in osteosarcoma specimens compared with normal bone tissue; (b) exogenous TSP‐2 enhanced osteosarcoma cell migration and levels of MMP‐9 expression; (c) TSP‐2‐induced increases were suppressed by siRNA knockdown of MMP‐9, αv,β3, PLCβ, PKCα or c‐Src and by treatment with inhibitors of PLCβ, PKCα, c‐Src, NF‐κB or IKK, or by DN mutants of IKKα and IKKβ; (d) exogenous TSP‐2 enhanced the phosphorylation levels of PLCβ, PKCα, c‐Src and NF‐κB and thereby increased their activities; (e) TSP‐2‐induced increases in NF‐κB activity are abolished by treatment with inhibitors of PLCβ, PKCα, c‐Src or NF‐κB and siRNA knockdown of PLCβ, PKCα or c‐Src, or by expressing DN‐mutant forms of IKKα and IKKβ; (f) TSP‐2 stimulation promoted the association of NF‐κB to the MMP‐9 promoter and subsequently up‐regulated MMP‐9 expression; and (g) osteosarcoma cells overexpressing TSP‐2 shRNA were less mobile; injection of these cells into mice resulted in fewer, smaller‐sized metastatic nodules compared with mice bearing tumours of vector control clones.

Our findings validate an association between TSP‐2 expression and osteosarcoma metastasis, underlying the importance of TSP‐2 as a specific marker for the progression and metastasis of osteosarcoma.

## 5 CONFLICT OF INTEREST

The authors declare no competing interests.

## AUTHOR CONTRIBUTION

**Ju‐Fang Liu:** Conceptualization (equal); Data curation (equal); Funding acquisition (equal); Writing‐original draft (equal); Writing‐review & editing (equal). **PoChun Chen:** Data curation (equal); Writing‐original draft (equal); Writing‐review & editing (equal). **Tsung‐Ming Chang:** Formal analysis (equal); Methodology (equal). **Chun‐Han Hou:** Conceptualization (equal); Data curation (equal); Funding acquisition (equal); Methodology (equal); Validation (equal); Writing‐original draft (equal); Writing‐review & editing (equal).

## Supporting information

Figures S1‐S2

## Data Availability

The data sets used and analysed during the current study are available from the corresponding author on reasonable request.
