# Supplementary material for: Thrombospondin‐2 stimulates MMP‐9 production and promotes osteosarcoma metastasis via the PLC, PKC, c‐Src and NF‐κB activation
Source: J Cell Mol Med. 2020 Oct 6;24(21):12826–39. doi: 10.1111/jcmm.15874 (PMC7686970; doi:10.1111/jcmm.15874)
Supplement: Supplementary file 1 — Figures S1‐S2 [file JCMM-24-12826-s001.pptx]

## Slide 1
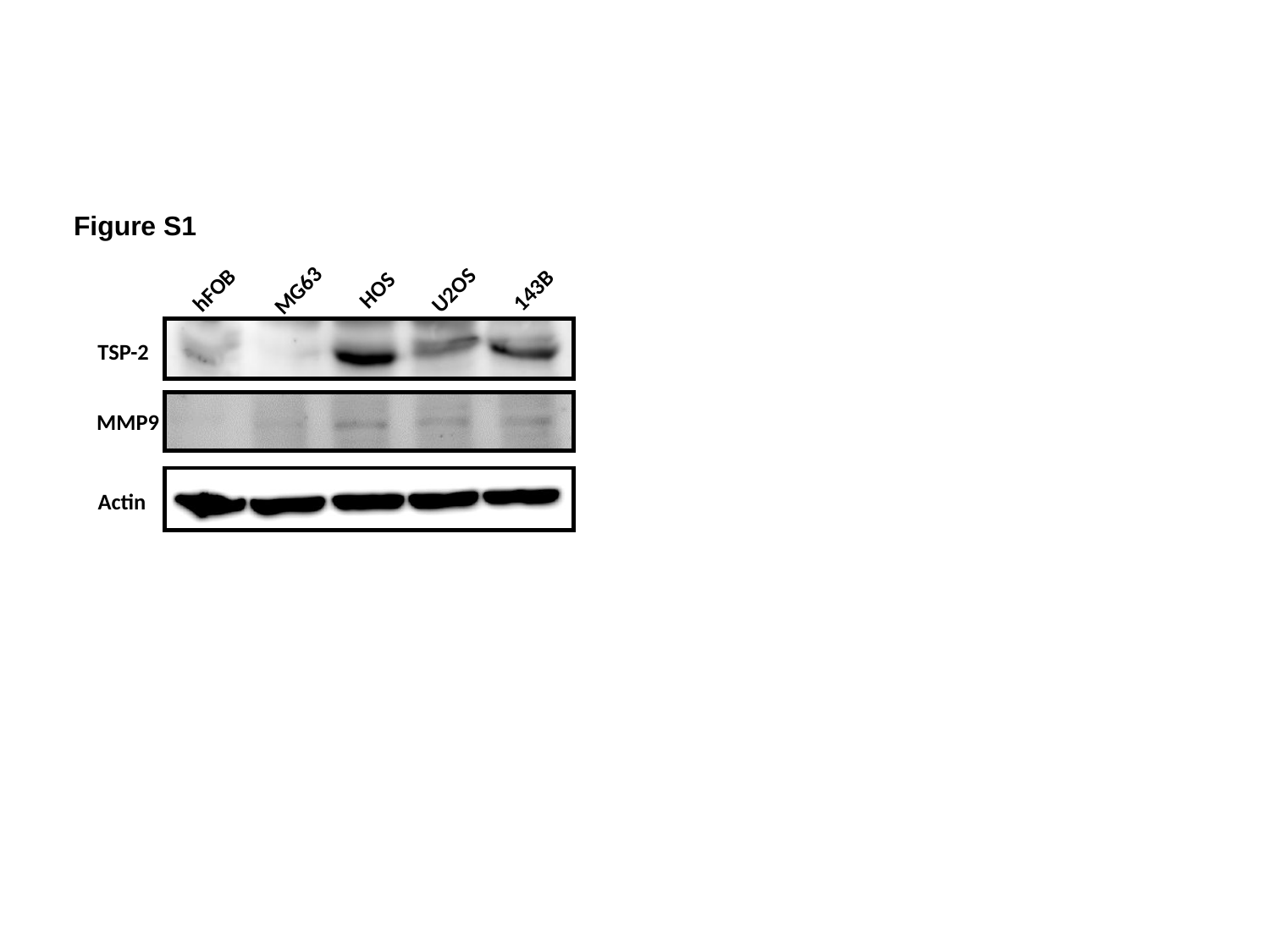

Figure S1
hFOB
MG63
HOS
U2OS
143B
TSP-2
MMP9
Actin

## Slide 2
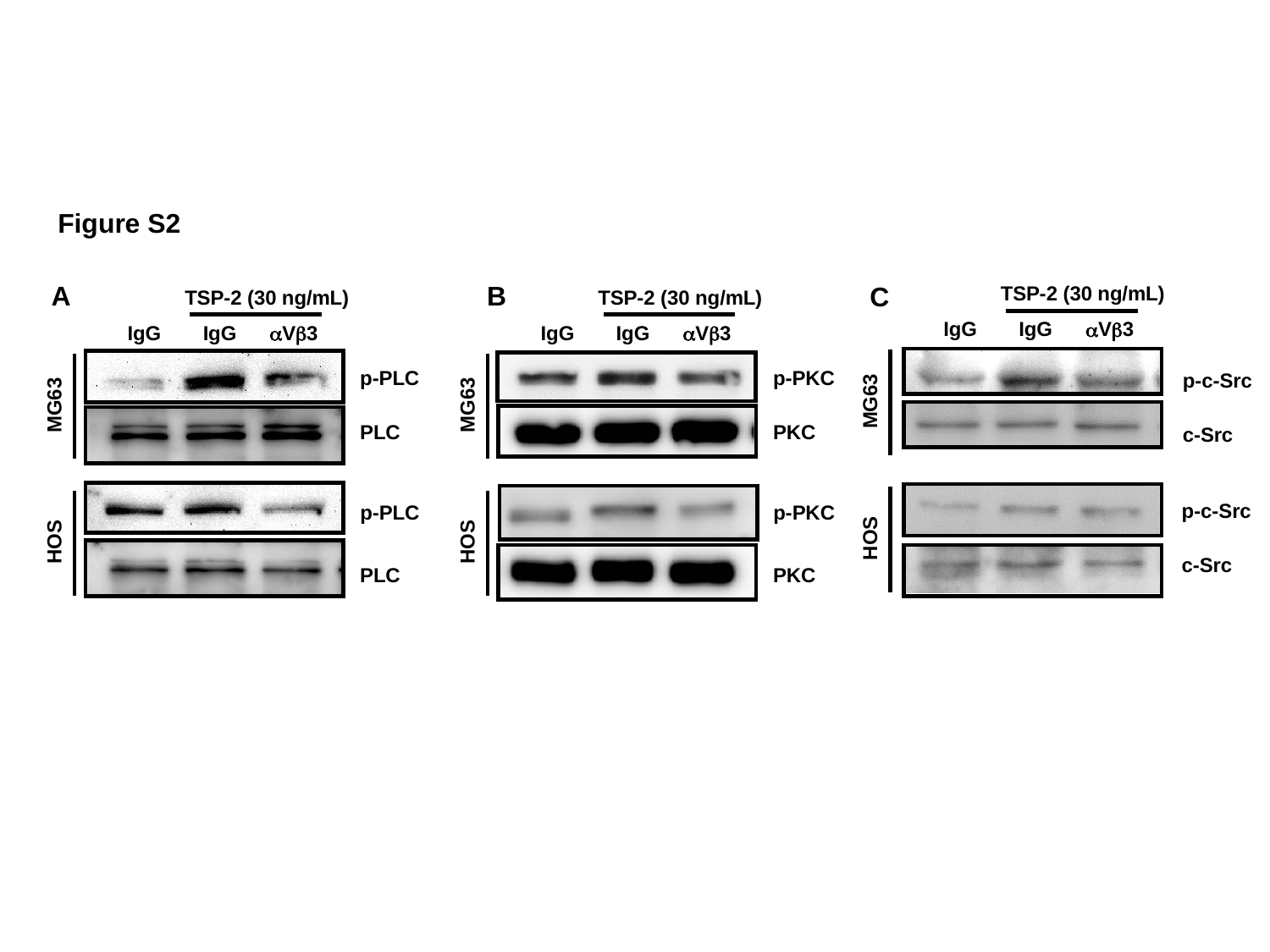

Figure S2
B
A
C
TSP-2 (30 ng/mL)
TSP-2 (30 ng/mL)
TSP-2 (30 ng/mL)
IgG
IgG
aVb3
IgG
IgG
aVb3
IgG
IgG
aVb3
p-PLC
p-PKC
p-c-Src
MG63
MG63
MG63
PLC
PKC
c-Src
p-c-Src
p-PLC
p-PKC
HOS
HOS
HOS
c-Src
PLC
PKC
